# Fibrothecoma of the Ovary; Clinical and Imaging Characteristics

**DOI:** 10.1089/whr.2024.0153

**Published:** 2025-03-25

**Authors:** Gehad Ahmad Saleh, Omar Hamdy, Dina Ragab, Bassante Farouk, Mennatalla Mahmoud Allam, Rawan Abo Asy, Fatmaelzahraa A. Denewar, Mohamed Ezat

**Affiliations:** ^1^ Radiology Department, Faculty of Medicine, Mansoura University, Mansoura, Egypt.; ^2^ Surgical Oncology Department, Oncology Center, Mansoura University, Mansoura, Egypt.; ^3^ Medical Intern, Mansoura University Hospitals, Mansoura, Egypt.

**Keywords:** fibrothecoma, sex cord-stromal tumor, ultrasound, CT, MRI

## Abstract

**Introduction:**

Ovarian fibrothecoma is a rare benign sex cord-stromal ovarian tumor sorted under the thecoma-fibroma group. We present an analysis of clinical and laboratory findings and the radiological characteristic features of pathologically proven fibrothecomas in variable imaging modalities.

**Methods:**

A retrospective analysis was done for 88 patients with 90 pathologically proven ovarian fibrothecoma between January 2011 and December 2023 from our center’s prospectively maintained database. All the patients underwent preoperative ultrasonography, computed tomography (CT), and magnetic resonance imaging (MRI) scans, clinical examinations, basic laboratory tests, and tumor markers.

**Results:**

The results of Spearman’s correlation revealed a statistically significant positive correlation between the largest tumor diameter and serum level. CA 125, the degree of ascites, and diffusion weighted imaging (DWI) signal intensity while the results of point biserial correlation revealed a statistically significant correlation of the largest tumor diameter with the presence of ascites, cystic changes, abdominal enlargement, surgery type, and border type. There were also statistically significantly higher hypoechoic lesions in the smaller tumor group (*p* = 0.001) but not for isoechoic (*p* = 0.099) and mixed (*p* = 0.052). Regarding the MRI, there was a statistically significantly larger tumor diameter in T2 mixed-hyperintense versus hypointense (*p* = 0.007) and intermediate (*p* = 0.010) signal intensities.

**Conclusion:**

Fibrothecoma showed a statistically significant positive correlation between the largest tumor diameter with serum level CA 125 and the amount of ascites. On imaging, it shows mild enhancement in both CT and MRI, with a statistically significant positive correlation of the largest tumor diameter with T2 and DWI signal intensity.

## Introduction

Ovarian fibrothecoma is a rare benign sex cord-stromal ovarian tumor sorted under the thecoma-fibroma group, that accounts for 4% of all ovarian cancers.^1^ The thecoma-fibroma group is composed of variable amounts of two components (theca cells and fibroblasts) and allocated into three subcategories fibromas, fibrothecomas, and thecomas, according to the proportion of the histological two-component.^2,3^ Fibromas rise from nonfunctioning fibroblast spindle cells of the ovarian stroma and, are frequently asymptomatic and encountered incidentally.^4^ Thecomas arise from theca cells and account only for 1% of all primary ovarian tumors. Thecomas may show signs of hyperestrogenism incorporating endometrial hyperplasia in 15% of cases.^5^ The term “fibrothecoma” refers to rare tumors with overlapping histological features and mixed spindle cells with lipid-rich cytoplasm (theca-like cells).^6–8^

Fibrothecoma typically is manifested unilaterally, but bilaterality could also occur.^9^ It can be presented with diverse clinical symptoms, and significant variations in tumor size, and internal components.^10^ It frequently occurs in perimenopausal and postmenopausal patients but can occur in younger women, with mean ages in the fifth and sixth decades.^3^ Fibrothecomas are most reported as solid masses, infrequently containing cystic components.^11^ Ovarian fibrothecomas may present with pleural effusions or ascites, known as Meigs’ syndrome.^12^

Common diagnostic means for this group include ultrasonography (US), computed tomography (CT), and magnetic resonance imaging (MRI).^2^ US is the first-line imaging tool for detecting ovarian abnormalities. However, sonographic characteristics of fibrothecoma are often unspecific.^5^ Despite the rise of CT utilization recently, ovarian tumors are frequently misdiagnosed due to their variable CT appearances and associated variable increased serum cancer antigen 125 (CA 125) levels as many tumors can be presented with large mass, cystic changes, and ascites.^13^ MRI has better soft tissue resolution and superiority in showing the distinct appearances of ovarian tumors.^14^ Diffusion weighted imaging (DWI) is a functional MRI sequence that helps adnexal lesion characterization and provides information about the tissue microenvironment.^14^ However, previous studies concerning MRI features of fibrothecoma had a small number of included cases.^15^

Due to the rarity of fibrothecoma, the previous studies on imaging features in the literature had relatively small sample sizes.^1,2,5,16^ Therefore, throughout this study, we present an analysis of clinical and laboratory findings, and the radiological characteristic features of pathologically proven fibrothecomas in variable imaging modalities aiming for reliable preoperative diagnosis to address this void.

## Patients and Methods

### Study population

The institutional review board granted this retrospective study, and a waiver of informed consent was received. This cohort study included patients diagnosed with pathologically proven fibrothecoma between January 2011 and December 2023 from our center’s prospectively maintained database. One hundred three females were initially evaluated for inclusion. The patients were referred to our center for preoperative assessment. Tumor characteristics were confirmed from the medical records after pathological examination. We excluded 15 patients with missed clinical and pathological data, the final study cohort consisted of 88 consecutive patients who underwent CT or MRI of the abdomen and pelvis within 2–3 weeks before surgery.

### Clinical and laboratory data

All the included patients underwent preoperative clinical examination, and basic laboratory tests (complete blood count, kidney and liver function test, coagulation profile, and virology screening and tumor markers: CA 125, carcinoembryonic antigen and CA 19-9. A pelvic ultrasound was performed as well as an anesthesia team consultation.

### Sonographic examination

Routine ultrasound examinations were performed with conventional transabdominal and transvaginal techniques. Also, doppler imaging was done with optimized parameters.

### CT technique

Contrast-enhanced CT scan was performed on a 128 multidetector CT scanner (GE Revolution). Scanning started from the level of the diaphragm to the symphysis pubis with the following CT scan acquisition parameters: 120 KVp, 220–400 mAs, a pitch of 1, section thickness of 5 mm, matrix 512 × 512, window width 400 and 0.5-seconds gantry rotation. Nonionic contrast medium (Omnipaque 350, GE Healthcare) was given at a 1.5 ml/kg dose with an injection rate of 3–4 mL/s. Image acquisition was attained with a delay of 70 second postcontrast injection. CT image raw data were then transferred onto a GE workstation for processing and reconstruction with a reconstruction slice thickness of 3 mm.

### MRI technique

MRI was performed using a 1.5-T MR scanner (Philips Ingenia, Best, Netherlands). Patients fasted for 4–6 hours before imaging. The scan range was from the umbilicus to the symphysis pubis. Imaging started with routine imaging sequences including T2 weight images (T2-WI) in the axial and sagittal planes with and without fat suppression, T1WI in the axial and sagittal plane with and without fat suppression. Diffusion-weighted images were acquired before contrast administration using an axial single-shot echo-planar sequence with *b* values (0, 500, 1000 s/mm^2^). The postcontrast images (three dimensional gradient echo sequence with fat saturation, THRIVE, Philips) were acquired after IV administration of Gadoterate meglumine (0.1–0.2 mmol/kg) by automatic injector followed by 10 ml saline infusion. MRI images were processed on an extended MR Workspace 2.6.3.5, Philips Medical Systems.

### Image interpretation

Image analysis was conducted by a consultant radiologist with 13 years of experience interpreting gynecological imaging. The images were assessed for the following features for each lesion: (1) the size (the maximum diameter in three orthogonal planes), (2) the location (right, left, or bilateral), (3) the tumor borders (smooth regular, lobulated or irregular), (4) presence of cystic changes, (5) US echogenicity (hypoechoic, isoechoic, mixed hyper-echoic), (6) US doppler vascularity, (7) MRI signal intensity characteristics on T2WI compared to the adjacent pelvic muscles (hypointense, isointense/intermediate, mixed hyper-intense), (8) DWI signal intensity was qualitatively assessed as low, intermediate or high signal similar for the pelvic bone, myometrium, and endometrium respectively. Corresponded apparent diffusion coefficient (ADC) maps were applicable using a Phillips Advantage Windows workstation to confirm restricted diffusion and differentiate it from the T2 Shine-Through Effect, (9) The degree of solid component enhancement was visually assessed on CT and MRI as mild (less than), moderate (equal) and marked (greater than) compared with the uterine myometrium enhancement, (10) functional estrogenic effect signs (endometrial thickening, uterine morphological changes), (11) degree of ascites (mild, moderate or marked), (12) other radiological signs (pleural effusion, and peritoneal disease).

### Surgical procedures

The multidisciplinary team assessed the patients and decided on the treatment plan according to imaging findings, and performance status. All the patients underwent surgical intervention through an open or laparoscopic approach. This was followed by either salpingo-oophorectomy or total abdominal hysterectomy and bilateral salpingo-oophorectomy according to the previous decision. The patients underwent routine postoperative care and were discharged once being fit for discharge.

### Pathological examination

The microscopic examination of the tumor was performed by expert pathologists, focusing on the tumor’s cellularity, fibrous component, and cystic degeneration. Routine hematoxylin and eosin staining was performed for every case and an immunohistochemical (IHC) examination was performed once indicated. Antibodies to smooth muscle actin, CD34, a-inhibin, vimentin, calretinin, and S-100 protein were used on need basis.

### Statistical analysis

Data were entered and analyzed using IBM-SPSS software (IBM Corp. Released 2020. IBM SPSS Statistics for Windows, Version 27.0. Armonk, NY: IBM Corp). Quantitative data were initially tested for normality using Shapiro-Wilk’s test with data being normally distributed if *p* > 0.050. The presence of significant outliers was tested by inspecting boxplots. Quantitative data were expressed as median and interquartile range. Categorical data for two groups were compared by chi-square test, Fisher’s exact test, and Fisher-Freeman-Halton exact test. Numerical data for two and multiple groups were compared by Mann-Whitney U test, and Kruskal-Wallis H test, respectively. Spearman’s correlation was used to assess the direction and strength of association between two numerical variables while point biserial correlation was used to assess the association between a numerical variable with a dichotomous variable. For any of the used tests, results were considered statistically significant if *p*-value ≤0.050. Appropriate charts were used to graphically present the results.

## Results

This study included 88 females with 90 ovarian fibrothecomas out of 1754 patients with ovarian tumors operated in this period. The clinical and radiological characteristics of the included patients are summarized in Table 1.

**Table 1.** The Clinical and Radiological Characteristics of the Included Patients

Characteristic	*N*	%
Presence of ascites	59	67
Degree of ascites		
Mild	46	78
Moderate	9	15.25
Marked	4	6.75
Associated adenomyosis	4	4.5
Associated atrophic endometrium	2	2.3
Associated endometrial hyperplasia	10	11.4
Associated uterine leiomyoma	2	2.3
Border		
Smooth (regular)	75	85.2
Lobulated	13	14.8
Clinical presentation		
Abdominal enlargement	6	6.8
Abnormal uterine bleeding	1	1.1
Asymptomatic (accidentally discovered)	3	3.4
Pelvic-abdominal pain	79	89.8
Abdominal	19	21.6
Pelvic	60	68.2
Cystic changes	57	64.8
Echogenicity by US (*n* = 86)		
Hypoechoic	41	47.7
Isoechoic	32	37.2
Mixed hyperechoic	13	15.1
Vascularity by US		
No	25	28.4
Mild	59	67
Moderate	3	3.4
Marked	1	1.1
CT enhancement	61	69.3
Mild	60	98.4
Moderate	1	1.6
MRI T2 signal intensity (*n* = 37)		
Hypointense	10	27
Intermediate/isointense	20	54.1
Mixed hyperintense	7	18.9
MR postcontrast enhancement (*n* = 37)		
Mild	35	94.6
Moderate	1	2.7
Marked	1	2.7
DWI signal intensity (*n* = 37)		
Low	8	21.6
Intermediate	27	73
High	2	5.4
Laterality		
Right	53	60.2
Left	33	37.5
Bilateral	2	2.3
Surgery type		
Salpingo-oophorectomy	52	59.1
Total abdominal hysterectomy & bilateral salpingo-oophrectomy	36	40.9
Surgical approach		
Open	83	94.3
Laparoscopic	5	5.7

Characteristic	Median	Q1–Q3
Age (years)	55	50–61
CA 125	80	29.7–291
CA 19-9	8.8	4.05–16
CEA	1	1–2
Largest tumor diameter (cm)	12	10–15

*N*, absolute frequency; Q1, 25th percentile; Q3, 75th percentile.

CA, cancer antigen; CEA, carcinoembryonic antigen; CT, computed tomography; MRI, magnetic resonance imaging; US, ultrasonography.

These characteristics were correlated with the maximum tumor diameter as described in Table 2 which shows the results of Spearman’s correlation revealing a statistically significant positive correlation of the largest tumor diameter with serum level of CA 125, the degree of ascites, and DWI signal intensity. It also shows the results of point biserial correlation which revealed a statistically significant correlation of the largest tumor diameter with ascites, cystic changes, abdominal enlargement, surgery type, and border type. There were no cases with cystic predominant fibrothecoma. The median of the largest tumor diameter was 13 cm versus 10 in those with and without ascites, 14 cm versus 11 in those with and without cystic changes, 19 cm versus 12 in those with and without abdominal enlargement, 12 cm versus 9 in open versus laparoscopic approach, and 20 cm versus 12 in those with lobulated versus smooth regular border.

**Table 2.** Correlations with the Tumor Maximum Diameter (cm)

Parameter	Correlation coefficient	Sig.
Spearman’s correlation		
Age (years)	0.064	0.551
CA 125	0.434	**<0.001**
CA 19-9	0.157	0.333
CEA	0.069	0.677
Vascularity (US)	0.121	0.261
MR postcontrast enhancement (*n* = 37)	−0.065	0.702
DWI signal intensity (*n* = 37)	0.457	**0.004**
CT enhancement	0.137	0.203
Degree of ascites	0.314	**0.016**
Point biserial correlation		
Presence of ascites	0.328	**0.002**
Presence of cystic changes	0.338	**0.001**
Presence of pelvic-abdominal pain	0.019	0.858
Presence of abdominal enlargement	0.304	**0.004**
Asymptomatic presentation	−0.205	0.056
Border	0.659	**<0.001**
Surgery type	−0.257	**0.016**
Surgical approach	0.083	0.444
Associated endometrial hyperplasia	0.141	0.192
Associated uterine leiomyoma	0.137	0.202
Associated adenomyosis	0.040	0.713
Associated atrophic endometrium	−0.031	0.773

Bold values indicate significant p values.

DWI, diffusion weighted imaging; Sig., significance (*p*-value).

There was a statistically significantly higher proportion of ascites in the larger versus smaller tumor group as shown in Table 3. All the 12 cases in the smaller tumor group had mild ascites. Most of the larger tumor group have mild ascites (34, 72.3%), 9 (19.1%) have moderate, and 4 (8.5%) have marked ascites. There was also a statistically significantly higher incidence of cystic changes, lobulated tumor borders, elevated CA 125 levels, and open versus laparoscopic surgery in the larger versus smaller tumor group.

**Table 3.** Comparisons of Larger (>10 cm) (*n* = 59) vs. Smaller (≤10 cm) Tumors (*n* = 29)

Parameter	Tumor group				Sig.
	Smaller		Larger		
Categorical	*N*	%	*N*	%	
Presence of ascites	12	41.4	47	79.7	**<0.001**
Laterality^a^					1.00
Right	17	58.6	36	61	
Left	11	37.9	22	37.3	
Bilateral	1	3.4	1	1.7	
Cystic changes	14	48.3	43	72.9	**0.023**
Border^b^					**0.004**
Regular smooth	29	100	46	78	
Lobulated	0	0	13	22	
Echogenicity (US)^a^					**0.003**
Hypoechoic	21	72.4	20	35.1	
Isoechoic	7	24.1	25	43.9	
Mixed hyperechoic	1	3.4	12	21.1	
Vascularity (US)^a^					0.461
No	11	37.9	14	23.7	
Mild	17	58.6	42	71.2	
Moderate	1	3.4	2	3.4	
Marked	0	0	1	1.7	
MRI T2 signal intensity^a^ (*n* = 37)					0.074
Hypointense	5	35.7	5	21.7	
Intermediate/isointense	9	64.3	11	47.8	
Mixed hyperintense	0	0	7	30.4	
MRI postcontrast enhancement^a^ (*n* = 37)					0.620
Mild	13	92.9	22	95.7	
Moderate	1	7.1	0	0	
Marked	0	0	1	4.3	
DWI signal intensity^a^ (*n* = 37)					**0.043**
Low	6	42.9	2	8.7	
Intermediate	8	57.1	19	82.6	
High	0	0	2	8.7	
CT enhancement^a^					0.207
No	12	41.4	15	25.4	
Mild	17	58.6	43	72.9	
Moderate	0	0	1	1.7	
Clinical presentation					
Abdominal enlargement^b^	0	0	6	10.2	0.172
Abnormal uterine bleeding^b^	1	3.4	0	0	0.330
Asymptomatic^b^	3	10.3	0	0	**0.033**
Pelvic-abdominal pain^b^	25	86.2	54	91.5	0.469
Abdominal	8	32	11	20.4	0.272
Pelvic	17	68	43	79.6	
Surgical technique					**0.039**
Open	25	86.2	58	98.3	
Laparoscopic	4	13.8	1	1.7	
Surgery type					0.075
Salpingo-oophorectomy	21	72.4	31	52.5	
TAH/BSO	8	27.6	28	47.5	
Associated endometrial hyperplasia^b^	2	6.9	8	13.6	0.487
Associated uterine leiomyoma^b^	0	0	2	3.4	1.00
Associated adenomyosis^b^	0	0	4	6.8	0.298
Associated atrophic endometrium^b^	1	3.4	1	1.7	1.00

Numerical	Median	Q1–Q3	Median	Q1–Q3	Sig.
Age (years)	55	46–60.5	55	50–62	0.631
CA 125	25	12.8–89.5	129.4	56.3–313	**<0.001**
CA 19-9	7.63	3.25–9.63	10.4	4.3–23.8	0.187
CEA	1	1–1.6	1.6	1–2.4	0.184

Bold values indicate significant p values.

Q1–Q3, 25th–75th percentiles; Sig., significance (*p*-value). The tests of significance for categorical data are chi-square test, Fisher’s exact test, and Fisher-Freeman-Halton exact test. The test of significance for numerical data is Mann-Whitney U test.

^a^Fisher-Freeman-Halton exact test.

^b^Fisher’s exact test.

A statistically significant difference in US echogenicity and DWI signal intensity (*p* = 0.003 and 0.043 respectively). So, dummy variables were created to compare each category versus the other two categories by performing multiple 2 × 2 Fisher’s exact tests with acceptance of statistical significance at *p* < 0.0167. This revealed statistically significantly higher hypoechoic lesions in the smaller tumor group (*p* = 0.001) but not for isoechoic (*p* = 0.099) and mixed hyper-echoic (*p* = 0.052), while no statistically significant differences in DWI low (*p* = 0.035), intermediate (*p* = 0.132), and high signal (*p* = 0.517).

**FIG. 1.**
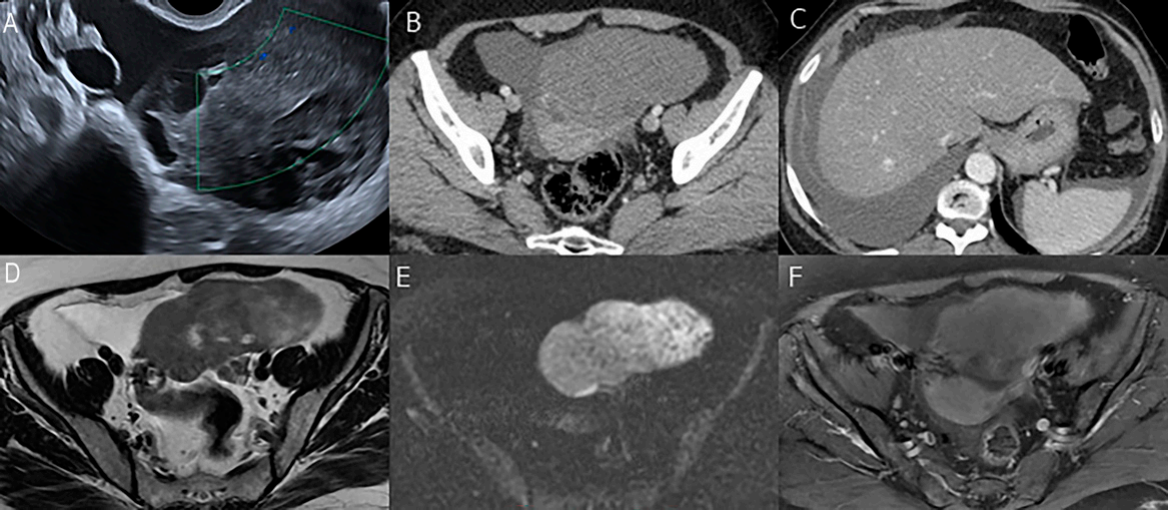
Left ovarian Fibrothecoma in a 55-year-old woman presented with pelvic pain. **(A)** Color Doppler transvaginal sonography shows an isoechoic lesion with faint vascularity. **(B** and **C)** Postcontrast CT axial images show a hypovascular left ovarian lesion with moderate ascites and right-sided pleural effusion (Meigs’ syndrome). **(D)** Axial T2-WI revealed a lobulated left ovarian solid lesion of predominant intermediate signal with focal slight hyperintensity and minute cystic changes. **(E)** Axial DWI (high *b* value = 1000) revealed a predominant intermediate signal. **(F)** Axial fat-suppressed postcontrast T1-WI revealed mild enhancement less than the myometrium.

**FIG. 2.**
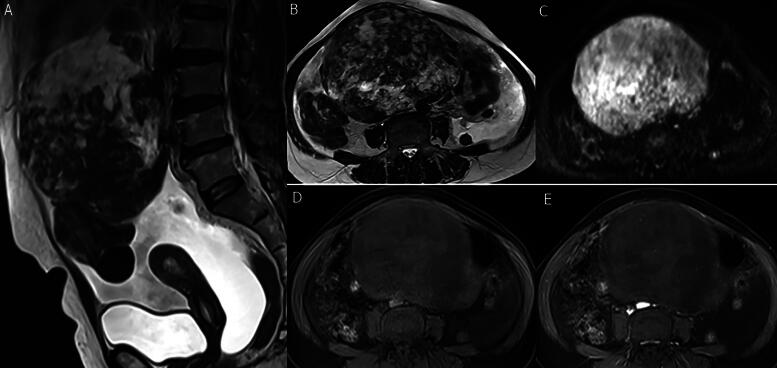
Huge right ovarian Fibrothecoma in a 67-year-old woman presented with abdominal enlargement. **(A** and **B)** sagittal and axial T2-WI revealed a large pelvi-abdominal lobulated mixed right ovarian lesion of mixed signal with predominant hyperintensity of cystic changes, associated endometrial thickening/hyperplasia, and marked ascites. **(C)** Axial DWI (high *b* value = 1000) revealed a predominant hyperintense signal (edema). **(D** and **E)** Axial fat-suppressed pre- and postcontrast T1 images revealed mild enhancement of the mass.

Furthermore, there was a statistically significant difference in the largest tumor diameter between the three types of US echogenicity, MR T2, and DWI signal intensity (Table 4). Pairwise comparisons revealed a statistically significantly larger tumor diameter in mixed hyperintense T2 signal versus hypointense (*p* = 0.007) and intermediate (*p* = 0.010). Pairwise comparisons with Bonferroni correction for multiple tests revealed a significantly lower largest tumor diameter in hypoechoic echogenicity versus mixed echogenicity (*p* = 0.001) and isoechoic (*p* = 0.004) but not between isoechoic and mixed hyper-echoic (*p* = 0.671). Pairwise comparisons with Bonferroni correction for multiple tests revealed a significantly lower largest tumor diameter in low DWI signal versus high DWI signal (*p* = 0.042) but not between low versus intermediate DWI signal (*p* = 0.095) and intermediate DWI versus high DWI signal (*p* = 0.424). Demonstrative cases are illustrated in Figures 1 and 2.

**Table 4.** Largest Tumor Diameter and MR T2 Signal Intensity, US Echogenicity

	*N*	Largest tumor diameter		Sig.
		Median	Q1–Q3	
MR signal intensity				
Hypointense	10	10	7–16	**0.005**
Intermediate/isointense	20	11	9.25–12	
Mixed hyperintense	7	16	16–20	
Echogenicity (US)				
Hypoechoic	41	10	8–13	**<0.001**
Isoechoic	32	14	11.25–15.75	
Mixed hyperechoic	13	16	12–19.5	
DWI signal intensity				
Low	8	8.5	7.25–11.5	**0.021**
Intermediate	27	12	10–16	
High	2	18	16–NA	

Bold values indicate significant p values.

Q1–Q3, 25th–75th percentiles; Sig., significance (*p*-value). The test of significance is Kruskal-Wallis H test.

NA, not available.

## Discussion

Our cohort showed a statistically significant positive correlation of the largest tumor diameter with serum level CA 125, the degree of ascites, and the cystic changes. Furthermore, there were statistically significant differences in the largest tumor diameter between US echogenicity, T2 signal intensity, and DWI signal intensity.

To begin with the clinical presentation, the most prevalent symptom in our study was pelviabdominal pain (89.8%). Nearly 50% of fibrothecoma cases produce estrogen, leading to occasional vaginal bleeding and irregular menstruation.^17^ However, these tumors typically do not significantly impact the patient’s overall health.^18^ Although 10 of the included patients had endometrial hyperplasia, only 1 case presented with abnormal uterine bleeding. This was comparable with the previously reported studies.^19,20^

Meigs’ syndrome is a benign infrequent condition with symptoms disappearing after the excision of the ovarian mass,^21^ only one of our cases had Meigs’ syndrome with right-sided pleural effusion and ascites. That was in line with multiple previous studies.^2,15,16^ In 90% of cases, ovarian fibrothecal tumors are unilateral and rarely malignant.^22^ In our study, none of the included patients have been diagnosed with malignant fibrothecoma, similar to other previous studies.^2,15^

In addition, our result revealed elevated serum CA 125 (>35 IU/mL) in 55 (65.5%) patients with a significantly higher proportion of higher CA 125 levels in the larger versus smaller tumor group. Similarly, previous studies showed elevated CA 125 in 28%.^16^ Tumor markers, most frequently CA 125, have been utilized to assess the malignancy of ovarian lesions that show up questionable on imaging. According to our findings, the rise of CA 125 does not mean malignant transformation, but it copes more with size enlargement or the presence of ascites or even pleural effusion (Meigs’ syndrome). That was in line with the previous studies.^23–29^

By immunohistochemical staining, the majority of fibrothecomas are positive for inhibin A, vimentin, Ki 67, and SMA. Endometrial hyperplasia, postmenopausal bleeding, increased serum CA 125 levels, and positive expression of Ki 67 are examples of estrogenic effects of fibrothecoma.^17^ In our cohort, the postoperative pathology revealed endometrial hyperplasia in 10 out of 36 patients who underwent hysterectomy.

One of the US hallmark findings of fibrothecoma is a round, oval, or lobulated hypoechoic mass with mild to moderate vascularity, while cystic or hemorrhagic changes are relatively rarely detected.^30^ In the US, fibrothecoma tumors appear as solid masses that are frequently mistaken for pedunculated subserous fibroid tumors.^31^ Most of our cases were hypoechoic (47.7%) and isoechoic (37.2%). Similarly, previous studies revealed the predominant hypo-echogenicity of fibrothecomas.^10,16^

Our results showed a statistically significant difference in US echogenicity with a significantly lower largest tumor diameter in hypoechoic echogenicity versus mixed hyperechogenicity (*p* = 0.001) and isoechoic (*p* = 0.004). Similarly, a previous study reported that large tumors demonstrated mixed hyperechogenicity.^10^

On CT imaging, fibrothecomas usually show solid tumor shapes, unilaterality, faint contrast enhancement, and no lymphadenopathy or peritoneal involvement, these criteria can help to exclude the diagnosis of other ovarian malignant masses.^32^ When fibrothecoma is associated with cystic changes and elevated serum level CA 125, it may be misdiagnosed as epithelial ovarian carcinoma.^23^ Our results revealed mild CT enhancement in 98.4% of patients, while only one patient showed moderate enhancement and none of the included patients showed marked enhancement. That agreed with previous studies.^1,2^

Regarding the MRI, fibrothecomas usually exhibit low signal intensity on T2-WI and mild postcontrast enhancement because of low blood flow, fibrotic content, and plenty of collagen.^33^ Fibrothecomas are hypovascular tumors, and central necrosis is partially caused by insufficient blood supply, as evidenced by the positive link between tumor growth and cystic degeneration.^17^ In previous reviews, MRI has been used to recognize up to 82% of fibrothecomas.^5,10,34^ If the fibrothecoma is estrogenically active, MRI imaging may be the primary means of demonstrating associated uterine changes such as uterine enlargement and endometrial thickening.^33^ The MRI features of fibrothecomas vary according to the lesion size, larger fibrothecomas are more likely to have degenerative changes, edema, heterogeneous mixed T2 signals, and heterogeneous enhancement.^5^ Other sex cord-stromal tumors could be differentiated from fibrothecoma by specific MRI criteria; granulose cell tumor may appear as a multilocular cystic mass with thick septa (Swiss cheese-sign) with marked restricted diffusion and intense postcontrast enhancement of both solid tissue and septae.^35^

This study revealed a statistically significant difference in the largest tumor diameter between the T2 signal intensities with a statistically significantly larger tumor diameter in masses of mixed hyperintense T2 signal. That was in concordance with the previous retrospective study conducted on 27 ovarian fibrothecomas, unlike our results, the correlation with the DWI signal intensity was not performed.^15^

DWI is a functional rapid MRI sequence that adds excellent tissue contrast relying on microscopic water motion (Brownian motion).^14,36^ The available data on the DWI characteristic of fibrothecoma is few. We focused on the qualitative signal assessment in our study as a previous study revealed no significant difference in quantitative ADC measurements.^37^ Most of our cases revealed a DWI intermediate signal (27/37, 73%) with a statistically significant positive correlation between the largest tumor diameter and DWI signal intensity. Similarly, a previous study on 18 fibrothecomas revealed that 61.1% (11/18) of lesions showed intermediate DWI signal intensity.^37^ Unlike our results, the correlation between the DWI signal and the largest tumor diameter was not performed.

Our study has limitations. First, it is a retrospective study. Second, the small number of the included patients. Third, pathology was described by a heterogeneous group of pathologists. Fourth, the patients did not undergo the same imaging modalities. But it has points of strength also, it presents a decade and a half experience of a tertiary referral center. It presents one of the largest reported series ever. Also, the imaging modalities were all reviewed by the same radiologist. Lastly, in view of being a benign disease, no long-term follow-up data is available.

## Conclusion

Fibrothecoma is a rare benign ovarian tumor that may be associated with ascites and elevated serum level CA 125. We found a statistically significant positive correlation between the largest tumor diameter with serum level CA 125 and the amount of ascites. On imaging, it shows mild enhancement in both CT and MRI, with a statistically significant positive correlation of the largest tumor diameter with T2 and DWI signal intensity.

## Data Availability

All the clinical, radiological, and pathological data used in this article are available on the Mansoura University medical system (Ibn Sina Hospital management system). http://srv137.mans.edu.eg/mus/newSystem/

## Abbreviations Used

ADC: apparent diffusion coefficient
CA: cancer antigen
CT: computed tomography
DWI: diffusion weighted imaging
IHC: immunohistochemical
MRI: magnetic resonance imaging
SMA: smooth muscle actin
US: ultrasound
vs: versus

## References

[1] Pat JJ, Rothnie KKM, Kolomainen D, et al. CT review of ovarian fibrothecoma. Br J Radiol 2022;95(1136):20210790; doi: 10.1259/bjr.20210790 35451310 PMC10162058

[2] Zhang Z, Wu Y, Gao J. CT diagnosis in the thecoma–fibroma group of the ovarian stromal tumors. Cell Biochem Biophys 2015;71(2):937–943; doi: 10.1007/s12013-014-0288-7 25315640

[3] Chen VW, Ruiz B, Killeen JL, et al. Pathology and classification of ovarian tumors. Cancer: Interdisciplinary International Journal of the American Cancer Society 2003;97(10 Suppl):2631–2642; doi: 10.1002/cncr.11345 12733128

[4] Javadi S, Ganeshan DM, Jensen CT, et al. Comprehensive review of imaging features of sex cord-stromal tumors of the ovary. Abdom Radiol (NY) 2021;46(4):1519–1529; doi: 10.1007/s00261-021-02998-w 33725145

[5] Shinagare AB, Meylaerts LJ, Laury AR, et al. MRI features of ovarian fibroma and fibrothecoma with histopathologic correlation. AJR Am J Roentgenol 2012;198(3):W296–W303; doi: 10.2214/AJR.11.7221 22358029

[6] Kurman RJ, Carcangiu ML, Herrington CS, et al. Classification of tumours of the ovary. WHO Classification of Tumours 2014;6:44–56.

[7] Roth LM, Czernobilsky B. Perspectives on pure ovarian stromal neoplasms and tumor-like proliferations of the ovarian stroma. Am J Surg Pathol 2011;35(3):e15–e33; doi: 10.1097/PAS.0b013e31820acb89 21317704

[8] Young RH. Ovarian sex Cord–Stromal tumors: Reflections on a 40-year experience with a fascinating group of tumors, including comments on the seminal observations of Robert E. Scully, MD. Arch Pathol Lab Med 2018;142(12):1459–1484; doi: 10.5858/arpa.2018-0291-RA 30500284

[9] Gupta A, Pathak S. Bilateral fibrothecoma: A rare case in young woman. Jcdr 2020; doi: 10.7860/jcdr/2020/43819.13680

[10] Chen H, Liu Y, Shen L, et al. Ovarian thecoma-fibroma groups: Clinical and sonographic features with pathological comparison. J Ovarian Res 2016;9(1):81–87; doi: 10.1186/s13048-016-0291-2 27876070 PMC5120502

[11] Jung SE, Rha SE, Lee JM, et al. CT and MRI findings of sex cord–stromal tumor of the ovary. AJR Am J Roentgenol 2005;185(1):207–215; doi: 10.2214/ajr.185.1.01850207 15972425

[12] Taylor EC, Irshaid L, Mathur M. Multimodality imaging approach to ovarian neoplasms with pathologic correlation. Radiographics 2021;41(1):289–315; doi: 10.1148/rg.2021200086 33186060

[13] Chung K-C, Lee HH, Su M-H, et al. Ovarian fibrothecoma mimicking ovarian cancer: Using laparoscopy to avoid unnecessary exploratory laparotomy. Taiwan J Obstet Gynecol 2019;58(6):903–904; doi: 10.1016/j.tjog.2019.09.026 31759556

[14] Tantawy MSI, Elrakhawy MM, El-Morsy A, et al. DWI in characterization of complex ovarian masses, would it help? The Egyptian Journal of Radiology and Nuclear Medicine 2018;49(3):878–883; doi: 10.1016/j.ejrnm.2018.01.006

[15] Wu B, Peng WJ, Gu YJ, et al. MRI diagnosis of ovarian fibrothecomas: Tumour appearances and oestrogenic effect features. Br J Radiol 2014;87(1038):20130634; doi: 10.1259/bjr.20130634 24670054 PMC4075549

[16] Yen P, Khong K, Lamba R, et al. Ovarian fibromas and fibrothecomas: Sonographic correlation with computed tomography and magnetic resonance imaging: A 5‐year single‐institution experience. J Ultrasound Med 2013;32(1):13–18; doi: 10.7863/jum.2013.32.1.13 23269706

[17] Chen J, Wang J, Chen X, et al. Computed tomography and magnetic resonance imaging features of ovarian fibrothecoma. Oncol Lett 2017;14(1):1172–1178; doi: 10.3892/ol.2017.6228 28693292 PMC5494683

[18] M K, Z TB, S DK, et al. Ovarian fibrothecoma: A case report. Saudi J Med Pharm Sci 2023;9(04):237–239; doi: 10.36348/sjmps.2023.v09i04.005

[19] Kazanci F, Arda İnan M, Erdem Ö, et al. Clinicopathological analysis and surgical approach of ovarian fibroma/fibrothecoma with 51 cases Opere edilen 51 fibrom/fibrotekom vakasının klinikopatolojik bulgularının değerlendirilmesi. ADYÜ Sağlık Bilimleri Derg 2021;7(3):176–182; doi: 10.30569.adiyamansaglik.877746

[20] Cho YJ, Lee HS, Kim JM, et al. Clinical characteristics and surgical management options for ovarian fibroma/fibrothecoma: A Study of 97 Cases. Gynecol Obstet Invest 2013;76(3):182–187; doi: 10.1159/000354555 24051436

[21] Yazdani S, Alijanpoor A, Sharbatdaran M, et al. Meigs’ syndrome with elevated serum CA125 in a case of ovarian fibroma/thecoma. Caspian J Intern Med 2014;5(1):43. 24490014 PMC3894471

[22] Kumari N, Bajaj B. Ovarian fibrothecoma—a diagnostic dilemma. Obstet Gynecol Int J 2019;10(3); doi: 10.15406/ogij.2019.10.00445

[23] Shen Y, Liang Y, Cheng X, et al. Ovarian fibroma/fibrothecoma with elevated serum CA125 level: A cohort of 66 cases. Medicine (Baltimore) 2018;97(34):e11926; doi: 10.1097/MD.0000000000011926 30142807 PMC6112998

[24] Toumi D, Mnejja A, Ghaddab I, et al. Challenging diagnoses in a case report: Ovarian fibrothecoma with elevated CA125 levels mimicking malignancy. Int J Surg Case Rep 2024;120:109847; doi: 10.1016/j.ijscr.2024.109847 38830334 PMC11180332

[25] Danilos J, Michał Kwaśniewski W, Mazurek D, et al. Meigs’ syndrome with elevated CA-125 and HE-4: A case of luteinized fibrothecoma. Prz Menopauzalny 2015;14(2):152–154; doi: 10.5114/pm.2015.52157 26327905 PMC4498034

[26] Yazdani S, Alijanpoor A, Sharbatdaran M, et al. Case report Meigs’ syndrome with elevated serum CA125 in a case of ovarian fibroma/thecoma. Caspian J Intern Med 2014(1):43–45. 5 24490014 PMC3894471

[27] Loué VAS, Gbary E, Koui S, et al. Bilateral ovarian fibrothecoma associated with ascites, bilateral pleural effusion, and marked elevated serum CA-125. Case Rep Obstet Gynecol 2013;2013(1):189072; doi: 10.1155/2013/189072 23431489 PMC3575674

[28] Sugiyama A, Urushihara N, Fukumoto K, et al. Ovarian fibroma with marked ascites and elevated serum levels of CA-125 in a young girl. J Pediatr Surg 2011;46(5):1001–1004; doi: 10.1016/j.jpedsurg.2011.02.054 21616270

[29] Yuan L, Cui L, Wang J, et al. A case report of Meigs’ syndrome caused by ovarian fibrothecoma with high levels of CA125. Int J Womens Health 2024;16(null):519–525; doi: 10.2147/IJWH.S450833 38544782 PMC10967536

[30] Jung SI. Ultrasonography of ovarian masses using a pattern recognition approach. Ultrasonography 2015;34(3):173–182; doi: 10.14366/usg.15003 25797108 PMC4484293

[31] Smorgick N, Maymon R. Assessment of adnexal masses using ultrasound: A practical review. Int J Womens Health 2014;6:857–863; doi: 10.2147/IJWH.S47075 25285023 PMC4181738

[32] Pat JJ, Rothnie KK, Kolomainen D, et al. CT review of ovarian fibrothecom. British Journal of Radiology 2022;95(1136); doi: 10.1259/bjr.20210790 PMC10162058 35451310

[33] Salemis NS, Panagiotopoulos N, Papamichail V, et al. Bilateral ovarian fibrothecoma. An uncommon cause of a large pelvic mass. Int J Surg Case Rep 2011;2(3):29–31; doi: 10.1016/j.ijscr.2010.07.005 22096681 PMC3199703

[34] Chechia A, Attia L, Temime RB, et al. Incidence, clinical analysis, and management of ovarian fibromas and fibrothecomas. Am J Obstet Gynecol 2008;199(5):473.e1–473.e4; doi: 10.1016/j.ajog.2008.03.053 18501324

[35] Zhang J, Zhang Y, Guo Y. Combination of clinical and MRI features in diagnosing ovarian granulosa cell tumor: A comparison with other ovarian sex cord-gonadal stromal tumors. Eur J Radiol 2023;158:110593; doi: 10.1016/j.ejrad.2022.110593 36434968

[36] Abdel Razek AAK, Elkalla HMHR, Refky B, et al. Assessment of tamoxifen-related endometrial changes in premenopausal female patients with diffusion-weighted magnetic resonance imaging. J Comput Assist Tomogr 2020;44(4):485–489; doi: 10.1097/RCT.0000000000001028 32558766

[37] Zhang H, Zhang G-F, Wang T-P, et al. Value of 3.0 T diffusion-weighted imaging in discriminating thecoma and fibrothecoma from other adnexal solid masses. J Ovarian Res 2013;6(1):58–59; doi: 10.1186/1757-2215-6-58 23962187 PMC3751813

